# Clinical characteristics and genetic testing outcome of suspected hereditary peripheral nerve sheath tumours in a tertiary cancer institution in Singapore

**DOI:** 10.1186/s13053-022-00230-4

**Published:** 2022-06-13

**Authors:** Jerold Loh, Pei Yi Ong, Denise Li Meng Goh, Mark E. Puhaindran, Balamurugan A. Vellayappan, Samuel Guan Wei Ow, Gloria Chan, Soo-Chin Lee

**Affiliations:** 1 Department of Haematology-Oncology, NCIS, National University Cancer Institute, Singapore, National University Health System, Singapore, Singapore; 2 Division of Paediatric Genetics and Metabolism, Department of Paediatrics, Khoo Teck Puat - National University Children’s Medical Institute, National University Hospital, Singapore, Singapore; 3 Department of Hand and Reconstructive Microsurgery, National University Hospital, Singapore, Singapore; 4 Department of Radiation Oncology, National University Hospital, Singapore, Singapore; 5 Cancer Science Institute, Singapore, Singapore

**Keywords:** Peripheral nerve sheath tumors, Schwannomatosis, Neurofibromatosis, LZTR1 variants, Genetics

## Abstract

**Background:**

Peripheral Nerve Sheath Tumors (PNST) are a diverse group of mostly benign tumours uncommon in the general population. About 5–10% of PNSTs are hereditary, predominantly arising from germline variants in *NF1, NF2, SMARCB1*, or *LZTR1* gene.

**Methods:**

We reviewed the clinical characteristics and genetic testing results of patients referred to the NCIS Adult Cancer Genetics Clinic for suspected hereditary PNST.

**Results:**

3,001 patients suspected to have various hereditary cancer syndromes were evaluated between year 2000 to March 2021. 13 (0.4%) were clinically diagnosed to have hereditary PNSTs. The majority were male (54%), with a median age at presentation to the genetics clinic of 29 years (range 19–48). 11/13 (85%) patients had multiple PNSTs, 12/13 (92%) had young onset PNSTs, 5/13 (38.5%) had personal and family history of PNST. 11/13 patients (85%) had clinical features of neurofibromatosis type 1 (NF1) including one patient who also fulfilled clinical criteria of neurofibromatosis type 2 (NF2); 2/13 (14%) had multiple schwannomas. Four patients underwent multi-gene panel testing, including one patient with clinical NF1, one patient who met both clinical NF1 and NF2 criteria, and two patients with multiple schwannomas. The patient with clinical features of NF1 was heterozygous for a pathogenic c. 2033dup variant in the *NF1* gene. The patient with both NF1/NF2 features was heterozygous for a novel c.732 T > A nonsense variant in the *NF2* gene. The two patients with multiple schwannomas were heterozygous for a pathogenic/likely pathogenic variant in the *LZTR1* gene and are the first *LZTR1*-positive schwannomatosis patients reported in Asia.

**Conclusion:**

Hereditary PNSTs are rare referrals to an adult cancer genetics clinic. NF1 is the most common PNST seen. LZTR1 variants may be the underlying cause in Asian patients with multiple schwannomatosis.

**Supplementary Information:**

The online version contains supplementary material available at 10.1186/s13053-022-00230-4.

## Introduction

Peripheral Nerve Sheath Tumors (PNST) are a mixed and diverse group of mostly benign tumours that are uncommon in the general population. Most do not have gender predilection, and age at presentation can be highly variable. Typical clinical presentations include a soft tissue mass, pain or focal neurological deficits due to mass effect or direct nerve invasion. The most common type of PNST is schwannoma followed by neurofibroma; other rare types of PNST include dermal nerve sheath myxoma, perineuroma and ganglioneuroma. Most PNSTs occur sporadically, with 90% of neurofibromas occurring in patients de novo [1].

Less than 5–10% of PNSTs have an underlying genetic condition. Those with a genetic syndrome are more likely to be diagnosed at a younger age, have multiple PNSTs, have a special subtype like plexiform neurofibromas, and/or have a positive family history. There are three major genetic syndromes associated with PNST tumours—Neurofibromatosis 1 (NF1), Neurofibromatosis 2 (NF2) and Schwannomatosis.

In adults ≥ 20 years old, NF1 is easily diagnosed clinically, using the National Institutes of Health (NIH) diagnostic criteria of the presence of at least two of the following features: six or more café au lait macules meeting size criteria, presence of two neurofibromas or one plexiform neurofibroma, axillary or inguinal freckling, optic glioma, Lisch nodules, bony dysplasias, and/or positive family history [2].

NF2 and schwannomatosis are more difficult to diagnose and distinguish clinically. The primary feature of NF2 is vestibular schwannomas, classically bilateral; other features include unilateral vestibular schwannomas, multiple meningiomas, ependymomas, juvenile cataracts, and a positive family history [3]. Schwannomatosis was only recognized as a clinically separate entity from NF2 in the late 1990s [4], upon identifying a subset of patients with multiple non-intradermal schwannomas but no vestibular schwannomas. Current clinical criteria identify patients with definite schwannomatosis when they are more than 30-years old and have all of the following features: two or more non-intradermal schwannomas with at least one histologically proven, no vestibular schwannomas, and does not meet NF2 diagnostic criteria nor have a first- degree relative with NF2 nor have a known NF2 variant [5]. However clinical lines are blurred between NF2 and schwannomatosis, as unilateral vestibular schwannomas have been reported in both conditions [6], as have other features like meningiomas [7].

NF1 and NF2 are due mostly to germline variants in the *NF1* and *NF2* genes respectively. With current testing methods, *NF1* likely pathogenic/pathogenic variants can be identified in around 95% of clinically diagnosed NF1 patients [8], while *NF2* likely pathogenic/pathogenic variants can be identified in around 60–93% of clinically diagnosed NF2 patients [9]. Schwannomatosis was first linked to the tumour suppressor gene *SMARCB1/INI1* [10] located on chromosome 22, which at present accounts for approximately 40–50% of familial schwannomatosis and 10% of sporadic cases [11, 12]. Analysis of *SMARCB1* variant-negative schwannomatosis patients led to the discovery of *LZTR1* variants in 2014 [13]. Much about LZTR1 variants remains unknown, with no published data in Asian patients. We describe a series of patients suspected to have hereditary PNSTs who were evaluated and tested at a Cancer Genetics Program at an academic cancer centre in Singapore.

## Material and methods

We reviewed patients who were referred to and evaluated at the National University Cancer Institute, Singapore (NCIS) Adult Cancer Genetics Clinic. We identified patients who were suspected to have PNST. These patients received genetic counselling and were offered clinical genetic testing using a multi-gene panel test that included the *NF1, NF2, SMARCB1,* and *LZTR1* genes. Full-gene sequencing and deletion/duplication analysis using next generation sequencing (NGS) technology were performed in clinical laboratories. Cascade testing was offered to first degree relatives in patients where relevant.

## Results

### Clinical features

Among the 3001 patients evaluated at the NCIS Cancer Genetics Clinic from year 2000 to March 2021, 13/3001 (0.4%) were patients who were referred for suspected hereditary PNSTs. The majority was male (54%). The median age at first presentation to the genetics clinic was 29 years (range 19–48). Majority of the patients (11/13) were referred from hospital specialists (surgeon = 5, pediatrician = 2, medical oncologist = 1, radiation oncologist = 1, dermatologist = 1, neurologist = 1), with the remaining 2 patients (15%) referred by primary care physicians. 11/13 (85%) patients had sufficient clinical features of NF1 to meet the NIH diagnostic criteria. One of these patients also met the clinical criteria for NF2. Two of 13 patients (15%) had multiple schwannomas without clinical features of NF1 and were suspected clinically to have NF2 or schwannomatosis. Detailed clinical features of patients are reflected in Table 1.

**Table 1:** .

Patient	Patient Profile	Age at presentation to genetics clinic	Referred by	Clinical Features (age at diagnosis)	Family History of PNST or related cancers or features suggestive of hereditary PNSTs	Family history of other unrelated cancers	Genetic Test Result		
							**Gene**	**Genetic Variant**	**Novel/Previously Reported**
1	Chinese Singaporean. Female	47	Breast Surgeon	5 Spinal Nerve Schwannomas along Cauda Equina, T9-10, L1-L2 (45–48), 1 right paraspinal nerve sheath Schwannoma (45)	Nil	Yes; Maternal aunt: benign brain tumor (40 s); maternal uncle: colorectal cancer (70 s); maternal first cousin: breast cancer (40 s); paternal uncle: liver cancer (60 s)	LZTR1	c.1768C > T; p.(Gln590*); Nonsense variant (pathogenic) Reference:NM_006767.3	Novel
2	Chinese Singaporean. Male	28	Radiation Oncologist	Left S2 nerve root schwannoma (27), multiple cauda equina schwannomas (27)	Nil	Nil	LZTR1	c.1210G > A; p.Gly404Arg; Possible splice site creation Reference:NM_006767.3	Previously reported (13, 21)
3	Chinese Singaporean Male	32	Musculoskeletal Oncologist	Single plexiform coccygeal nerve neurofibroma (33); > 5 café au lait macules > 15 mm	Nil	Nil	NF1	c.2033dup; p. Ile679Aspfs*21; Duplication (pathogenic). Reference:NM_000267.3	Previously reported (14–18)
4	Chinese Singaporean. Female	22	Hand Surgeon	Cutaneous Neurofibromas, > 5 café au lait macules > 15 mm, axillary freckling, L1-L4 intradural extramedullary neurogenic tumour, large right C2 extradural neurogenic tumour, Bilateral acoustic neuroma with brainstem compression, Bilateral trigeminal nerve schwannomas, Bilateral jugular foramen neurofibroma (22)	Nil	Yes; Mother: Breast cancer (46), 4 Maternal aunts: Breast cancer (ages 58, 64, 66 and 66), Maternal first cousin: Breast cancer (37), Maternal grandfather: Brain tumor (60 s)	NF2	c.732 T > A; p. Tyr244Ter; Nonsense variant (likely pathogenic). Reference:NM_016418.5	Novel
5	Chinese Singaporean Male	32	Dermatologist	Right ankle deep peroneal nerve plexiform neurofibroma (27); left brachial plexus plexiform neurofibroma (32); multiple cutaneous neurofibromas; axillary freckling, multiple bilateral Lisch nodules, > 5 café au lait macules > 15 mm	Nil	Nil	-	Not done	-
6	Chinese Singaporean Female	48	Neurologist	Multiple cutaneous neurofibromas, axillary freckling, bilateral multiple Lisch nodules, > 5 café au lait macules > 15 mm	Yes; Mother: clinical features of NF-1; maternal grandmother: clinical features of NF-1	Yes; daughter: leukemia (3), sister blood cancer (18)	-	Not done	-
7	Indian Singaporean. Male	37	Pediatrician	Multiple cutaneous and subcutaneous neurofibromas, axillary freckling, > 5 café au lait macules > 15 mm	Yes; son: clinical features of NF-1	Yes; mother: colorectal cancer (50 s), paternal uncle: lung cancer (56); maternal first cousin: breast cancer (40 s); maternal great grandfather: throat cancer (unknown age)	-	Not done	-
8	Chinese Singaporean Male	19	Primary care physician (military screening)	Right lumbosacral paravertebral nerve plexiform neurofibroma (19), axillary freckling, > 5 café au lait macules > 15 mm	Yes; Brother: clinical features of NF-1; mother: clinical features of NF-1	Nil	-	Not done	-
9	Chinese Singaporean Female	22	Pediatric Nephrologist	Multiple cutaneous neurofibromas, axillary freckling, > 5 café au lait macules > 15 mm	Yes; Father: clinical features of NF-1; sister: clinical features of NF-1	Nil	-	Not done	-
10	Chinese Singaporean Female	29	Musculoskeletal Oncologist	Left tibial nerve plexiform neurofibroma (27), multiple cutaneous neurofibromas, > 5 café au lait macules > 15 mm	Nil	Yes; Father: prostate cancer (50 s)	-	Not done	-
11	Chinese Singaporean Female	18	Primary care physician	Multiple cutaneous neurofibromas, axillary freckling, > 5 café au lait macules > 15 mm	Yes; Father: clinical features of NF-1; paternal grandmother: clinical features of NF-1	Nil	-	Not done	-
12	Chinese Singaporean Male	40	Medical Oncologist	Malignant PNST (right medial thigh, 39), multiple cutaneous neurofibroma, axillary freckling	Nil	Nil	-	Not done	-
13	Chinese Singaporean Male	21	Musculoskeletal Oncologist	Plexiform neurofibroma of right superficial branch of radial nerve (since birth), multiple cutaneous neurofibromas, > 5 café au lait macules > 15 mm, axillary freckling	Nil	Yes; Mother: cervical squamous cell carcinoma (43)	-	Not done	-

### Genetic testing and case description of positive cases

Four of the 13 patients underwent germline genetic testing (31%): 2/2 patients with suspected schwannomatosis, 2/11 patients with clinically diagnosed NF1; including the patient that met clinical criteria for both NF1 and NF2. Most clinically suspected NF1 patients declined genetic testing as they felt that genetic information would not change their diagnosis and clinical management.

**Fig. 1:**
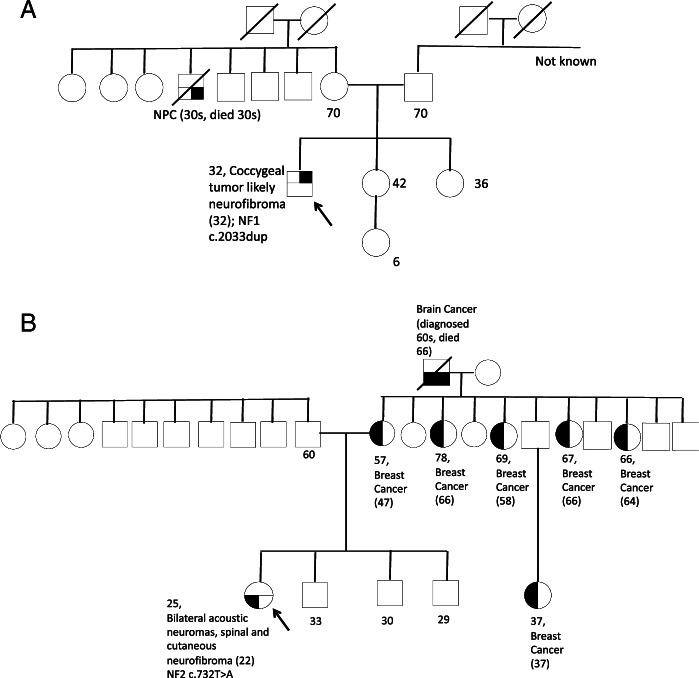
**A** Genogram, Table 1 Patient 3, **B** Genogram, Table 1 Patient 4

One of the clinically diagnosed NF1 (Patient Three) who underwent genetic testing was heterozygous for a pathogenic frameshift *NF1* variant. (Table 1 & Fig. 1A). He presented with café au lait macules and an asymptomatic coccygeal plexiform neurofibroma incidentally picked up on imaging at age 32 to evaluate for male urinary tract infection. He was heterozygous for a known pathogenic frameshift variant (c.2033dup) in the *NF1* gene, which has been previously reported in NF1 patients (14–18). He did not have family history of NF-1, suggesting that his NF1 variant was de novo.

The patient who met clinical criteria for both NF1 and NF2 (Patient Four) tested heterozygous for a novel likely pathogenic *NF2* nonsense variant. (Table 1 & Fig. 1B). She presented at age 22 with unsteady gait and bilateral sensorineural hearing loss. This was found to be due to bilateral acoustic neuroma with brainstem compression for which she underwent craniotomy and debulking of the left vestibular schwannoma. She also had extensive neurogenic tumours at multiple cord levels (right C2 extradural region, L1-L4 intradural region), as well as neurofibromas involving bilateral trigeminal nerves and neurofibromas in the jugular foramen, extensive cutaneous neurofibromas and café au lait macules. She was heterozygous for a pathogenic nonsense variant in the *NF2* gene (*c.732 T* > *A,* pTyr244Ter). No variants in *NF1, SMARCB1,* or *LZTR1* were identified. The variant has not been reported in any population databases. The patient did not have any family history of neurofibromatosis but did have a strong family of cancer. Her mother was diagnosed with breast cancer at age 46, and she has four maternal aunts who were diagnosed with breast cancer at ages 58, 64, 66 and 66 respectively. A maternal cousin was also diagnosed with breast cancer at age 37 and her maternal grandfather was diagnosed with brain tumor in his 50 s.

**Fig. 2:**
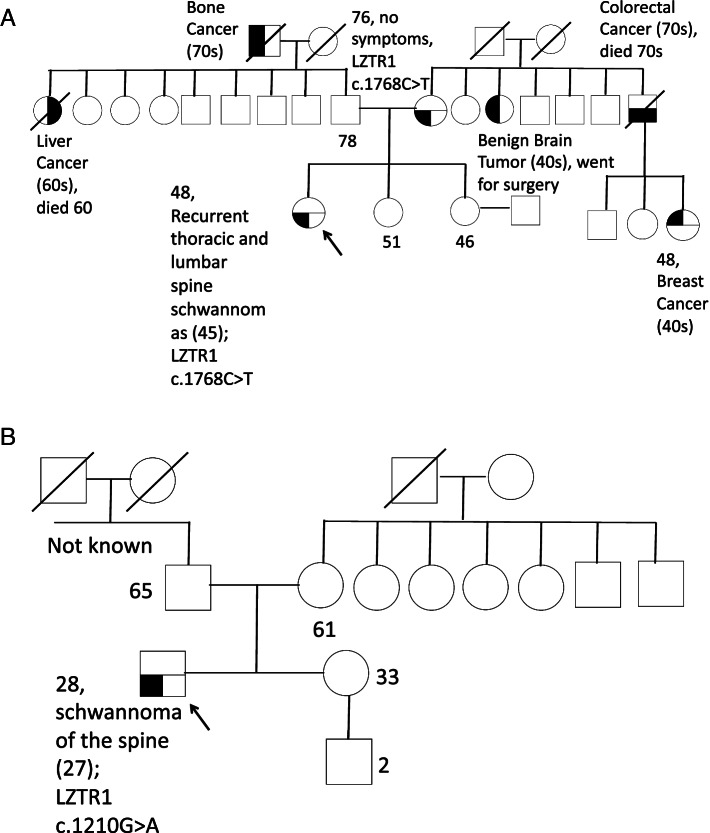
**A** Genogram, Table 1 Patient 1, **B** Genogram, Table 1 Patient 2

Both patients with multiple schwannomas (Patient One and Two) were heterozygous for *LZTR1* variants. Patient One (Table 1 & Fig. 2A) is a 48-year-old Chinese female, who presented with lower back pain secondary to a T10 schwannoma at age 45. The tumor was associated with multiple enhancing nodular lesions along the surface of the cauda equina and right paraspinal region suggestive of nerve sheath tumours. It was excised, and the histology showed spindle cell tumours with fascicular architecture, with focal characteristic anuclear zones with palisading spindle cell nuclei staining strongly positive for S100, in keeping with schwannomas. At 47, she developed recurrence of back pain and lower limb numbness, and this was found to be due to new T9 and L1 schwannomas. She underwent surgery, and the histology similarly was consistent with schwannomas. The patient did not have any clinical features suggestive of NF1. An MRI of the brain did not show any vestibular schwannomas. The patient had no known family history of schwannomas or cutaneous lesions suggestive of NF1. A maternal aunt had a history of a benign brain tumour at age 40 s which was surgically excised. Her mother was asymptomatic and had no history of cutaneous lesions or PNSTs. Clinically, the patient fulfilled the criteria for definite schwannomatosis.

The patient underwent multigene testing and was found to be heterozygous for a novel pathogenic *LZTR1* nonsense variant (*c.1768C* > *T*, p. Gln590*). No variants in *NF1, NF2,* or *SMARCB1* were identified. Her parents and elder sister underwent genetic counseling and cascade testing. Her mother tested positive for the same *LZTR1* variant, confirming the maternal origin of the variant. Her father and sister tested negative for the *LZTR1* variant.

Patient Two (Table 1 & Fig. 2B) is a 28-year-old Chinese man who presented at the age of 25 with back pain. Workup eventually showed him to have a sacral schwannoma located at S2 exit neural foramen. The tumor was associated with multiple subcentimetre enhancing nodules along the cauda equina suspicious of neurogenic tumors. An MRI of the brain did not show any vestibular schwannomas. He had no family history of PNST, cutaneous neurofibromas or cancer. The tumour was excised and the histology was in keeping with schwannoma as above. Based on this, the patient fulfilled clinical criteria for possible schwannomatosis.

Genetic testing revealed that he was heterozygous for a missense *LZTR1* variant, (*c.1210G* > *A*, p.Gly404Arg). No variants in *NF1, NF2*, or S*MARCB1* were identified. No family testing was done based on family preference. The *LZTR1* variant replaced glycine with arginine; the glycine residue is highly conserved, and there is a moderate physicochemical difference between glycine and arginine. This is reported to affect LZTR1 protein function [19]. Algorithms developed to predict the effect of sequence changes on RNA splicing suggested that this variant may create or strengthen a splice site, although the prediction had not been confirmed by published transcriptional studies. It is thus currently labelled by the testing lab as a variant of unknown significance (VUS). However, based on the American College of Medical Genetics (ACMG) variant classification, this missense variant is likely pathogenic (PS4, PM2, PM6). [20] This is because the variant was not present in population databases, but had been observed in individuals with schwannomatosis (PS4) [13, 21]. It also results in protein length changes as a result of in-frame mutations (PM2) and it is a suspected *de-novo* mutation, although without paternal or maternal confirmation (PM6).

## Discussion

Of the patients who underwent genetic testing in our series, most of the diagnosis based on genetic testing was concordant with that made based on clinical criteria. One of our patients fulfilled clinical criteria of both NF1 and NF2, and genetic testing was key to elucidate the underlying diagnosis. This highlights the utility of genetic testing in cases where clinical features do not fulfill clinical diagnostic criteria or fulfill multiple criteria. Identifying the causative genetic variant can facilitate testing and screening asymptomatic family members, which was the reason one of our patients chose to pursue genetic testing, as well as providing definitive diagnostic evidence some patients require to comply with surveillance.

Among the various forms of hereditary PNSTs, NF1 is the commonest cause, with an estimated incidence of approximately 1:2600 to 3000 [22]. It is an autosomal dominant condition arising from pathogenic and likely pathogenic variants in the *NF1* gene, located at chromosome 17q11.2 [23], resulting in reduced production or function of neurofibromin, which works to inhibits the Ras p21 mitogenic signaling pathway. The usual order of appearance of clinical features is café-au-lait macules, axillary freckling, Lisch nodules, and neurofibromas [24].

Due to high allelic heterogeneity in NF1, there are few genotype–phenotype correlations seen. Patient Three who presented with café au lait macules and young-onset plexiform neurofibroma at age 32 was heterozygous for a pathogenic *NF1* frameshift variant (c.2033dup) that has previously been reported in multiple individuals with NF-1 [14–18] around the world, including from Asia.

NF2 has an estimated incidence of 1:60 000 [25] and is also an autosomal dominant condition arising from variants in the *NF2* gene, located on chromosome 22, resulting in reduced production of the protein schwannomin which acts as a tumour suppressor [26]. Patient Four presented with multiple symptomatic intracranial and spinal neurogenic tumors including bilateral acoustic neuromas and was heterozygous for a likely pathogenic *NF2 c.732 T* > *A* (p.Tyr244*) nonsense variant. This variant was believed to truncate the NF2 protein causing loss-of-function and was likely pathogenic. It was novel and has not been reported in general population databases (1000 Genomes Project, Exome Variant Server, and Genome Aggregation Database), ClinVar or COSMIC databases.

Schwannomatosis has an estimated prevalence of 1: 126 000 [6], and causative genes include *SMARCB1* and *LZTR1*. The median age of symptom onset is around 30 years and median age of diagnosis around 40 years, with no predilection of gender or race [27]. Pain is the most common presenting complaint, with chronic pain affecting up to 60% of patients in some series. The nature of pain is complex and may not always be associated with a mass. Palpable masses are also a common presenting symptom in around 40% of patients. The hallmark feature is multiple schwannomas, which most often originate from peripheral nerves in the arms or legs, but can also be found in the head, neck or trunk. Spinal nerve root schwannomas are common, occurring in 75% in some series, with the lumbar spine being the most common location and can frequently be multifocal [27]. These usually arise from dorsal sensory roots and hence can present with sensory changes. Subcutaneous schwannomas occur in 20–30% and cranial nerve schwannomas in 10% of patients [27, 28].Schwannomatosis patients are at increased risk of other tumours like meningioma, malignant PNST and rhabdoid tumours [27, 29, 30].

*SMARCB1* variant-positive schwannomatosis is thought to involve a four-hit, three-step model of tumorigenesis, where the initial SMARCB1 variant triggers partial loss of chromosome 22 containing the wildtype *SMARCB1* and *NF2*, and finally followed by a spontaneous mutation in the remaining wildtype *NF2* [31].

*LZTR1* is a tumor suppressor gene, which codes for one of the BTB-Kelch group of proteins. It contains two functional domains with a Kelch-BTB-BACK-BTB-BACK motif. The BTB domains interact with cullin 3 (CUL3)-RING ubiquitin ligase (CRL3) complex, which engages in protein ubiquitination, including those involved in mitogenic pathways like RAS [19, 32]. Hence *LZTR1* loss results in enhanced RAS activity and downstream mitogenic signaling, with increased growth in cellular models [32]. Like *SMARCB1*, a spontaneous mutation in *LZTR1* leads to a similar four-hit, three-step model to tumorigenesis.

*LZTR1* variants have been reported in 26–80% of SMARCB1 variant-negative schwannomatosis patients [13, 33]. At the point of writing, there are fewer than 150 patients world-wide with confirmed pathogenic *LZTR1* variants reported in the literature, with most studies in the United States and parts of Europe (France, Netherlands, Italy, Spain) [13, 32–40]. To the best of our knowledge, our two patients represent the first reported cases of *LZTR1* related schwannomatosis from Asia. *LZTR1* related schwannomatosis has been reported to be more associated with spinal schwannomatosis [37] and unilateral vestibular schwannomas [33], and with pain being the main presenting complaint [37]. The presentation of the two patients in our series was consistent with this, with both having multiple spinal schwannomas and chronic pain.

There have only been a handful of case reports of clinical schwannomatosis in Asia [41–45], owing to its rarity but also likely under-recognition. Most of the reported cases were suspected from clinical criteria without confirmatory genetic testing. One patient from Japan presenting with a left intraorbital schwannomas and multiple spinal schwannomas was tested and found to be *SMARCB1* and *LZTR1* negative [43], while another Japanese family – father and son pair with the father presenting with thoracic spinal and cutaneous schwannomas and his 35-year old son with a left cerebropontine angle schwannoma—were found to carry *SMARCB1* pathogenic variants [41].

To the best of our knowledge, we report the first two unrelated patients in Asia with schwannomatosis attributed to pathogenic or likely pathogenic *LZTR1* variants. Both patients are Chinese. Patient One who presented with multiple spinal schwannomas at age 45 was heterozygous for an *LZTR1* nonsense variant (c.1768C > T; p.Gln590*) that has not been previously reported. Interestingly, while the patient reported no family history of schwannomatosis, subsequent cascade testing revealed her 76-year old asymptomatic mother to be a carrier, suggesting incomplete penetrance. Incomplete penetrance was also observed in other reports [13, 33, 40, 46] although exact degree of penetrance of *LZTR1* gene is still unknown. Patient Two was heterozygous for a *LZTR1* missense variant (c.1210G>A; p. Gly404Arg) that may create or strengthen a splice site; this variant has previously been reported in two patients with schwannomatosis from the United States, including a 70-year old male who presented with a right vestibular schwannoma at age 34, two thoracic spinal schwannomas removed at age 43, and multiple cranial nerve schwannomas since age 55 [21]; he fulfilled Manchester criteria for clinical diagnosis of NF2 but was found instead to carry an LZTR1 variant. [13, 21].

Our study has several limitations. Firstly, not all patients with PNST may have been referred to the adult cancer clinic. Hence our reported incidence may not be reflective of the true population incidence. Secondly, the duration of our study spanned 17 years, from the time when genetic testing was not widely available to the current day’s situation when genetic testing is much more accessible. Hence the uptake rate of genetic testing may not be a true reflection of the current state. Furthermore, LZTR1 as a gene predisposing to hereditary schwannomas was only known since 2014 and hence may not be tested in patients who underwent genetic testing prior to that.

## Conclusion

Hereditary PNSTs are rare referrals to an adult cancer genetic clinics accounting for less than 1% of all referrals. NF1 was the most commonly encountered cause, and the diagnosis was made clinically in all patients. We report the first two Asian patients with schwannomatosis due to pathogenic or likely pathogenic *LZTR1* variants.

## Supplementary Information

**Additional file 1: Figure 1A. **Genogram, Table 1 Patient 5.** Figure 1B. **Genogram, Table 1 Patient 6.** Figure C. **Genogram, Table 1 Patient **7. Figure 1D.** Genogram, Table 1 Patient 8.** Figure 1 E. **Genogram, Table 1 Patient 9.

## Data Availability

Data sharing is not applicable to this article as no datasets were generated or analysed during the current study.
