# Supplementary material for: Clinical characteristics and genetic testing outcome of suspected hereditary peripheral nerve sheath tumours in a tertiary cancer institution in Singapore
Source: Hered Cancer Clin Pract. 2022 Jun 13;20:23. doi: 10.1186/s13053-022-00230-4 (PMC9195433; doi:10.1186/s13053-022-00230-4)
Supplement: Supplementary file 1 — Additional file 1: Figure 1A. Genogram, Table 1 Patient 5. Figure 1B. Genogram, Table 1 Patient 6. Figure C. Genogram, Table 1 Patient 7. Figure 1D. Genogram, Table 1 Patient 8. Figure 1 E. Genogram, Table 1 Patient 9. [file 13053_2022_230_MOESM1_ESM.pptx]

## Slide 1
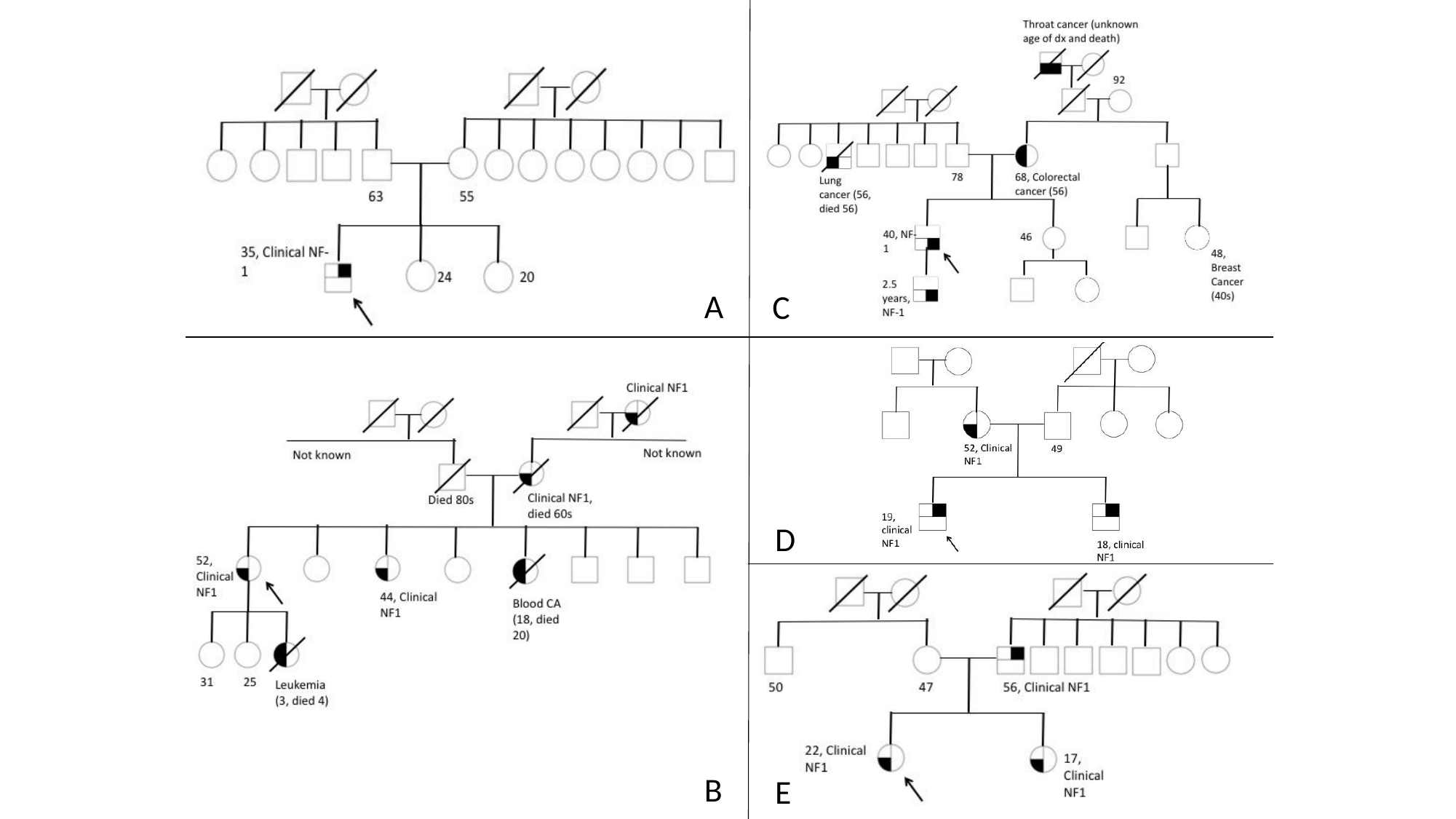

A
C
D
B
E

## Slide 2
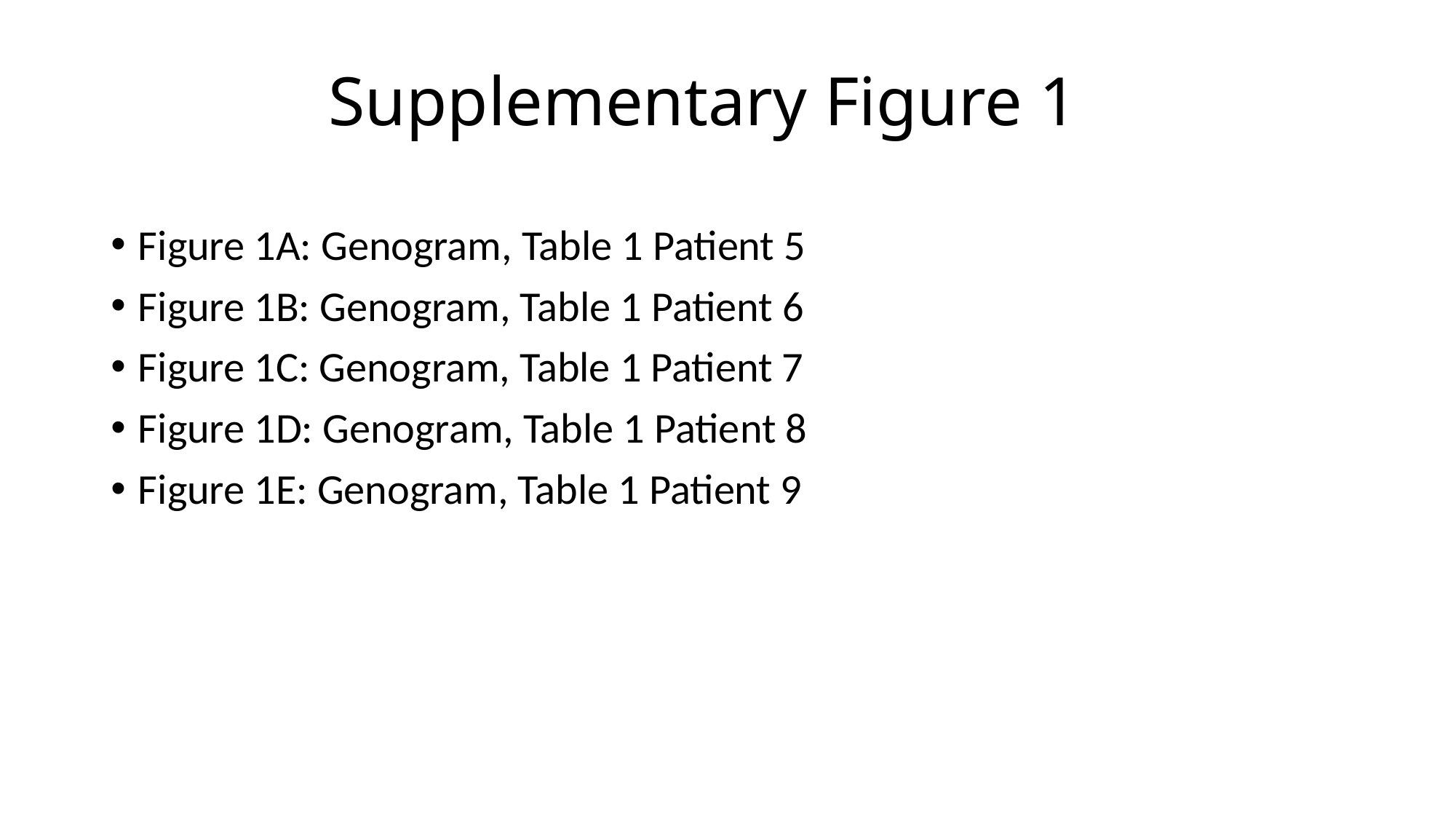

# Supplementary Figure 1
Figure 1A: Genogram, Table 1 Patient 5
Figure 1B: Genogram, Table 1 Patient 6
Figure 1C: Genogram, Table 1 Patient 7
Figure 1D: Genogram, Table 1 Patient 8
Figure 1E: Genogram, Table 1 Patient 9

## Slide 3
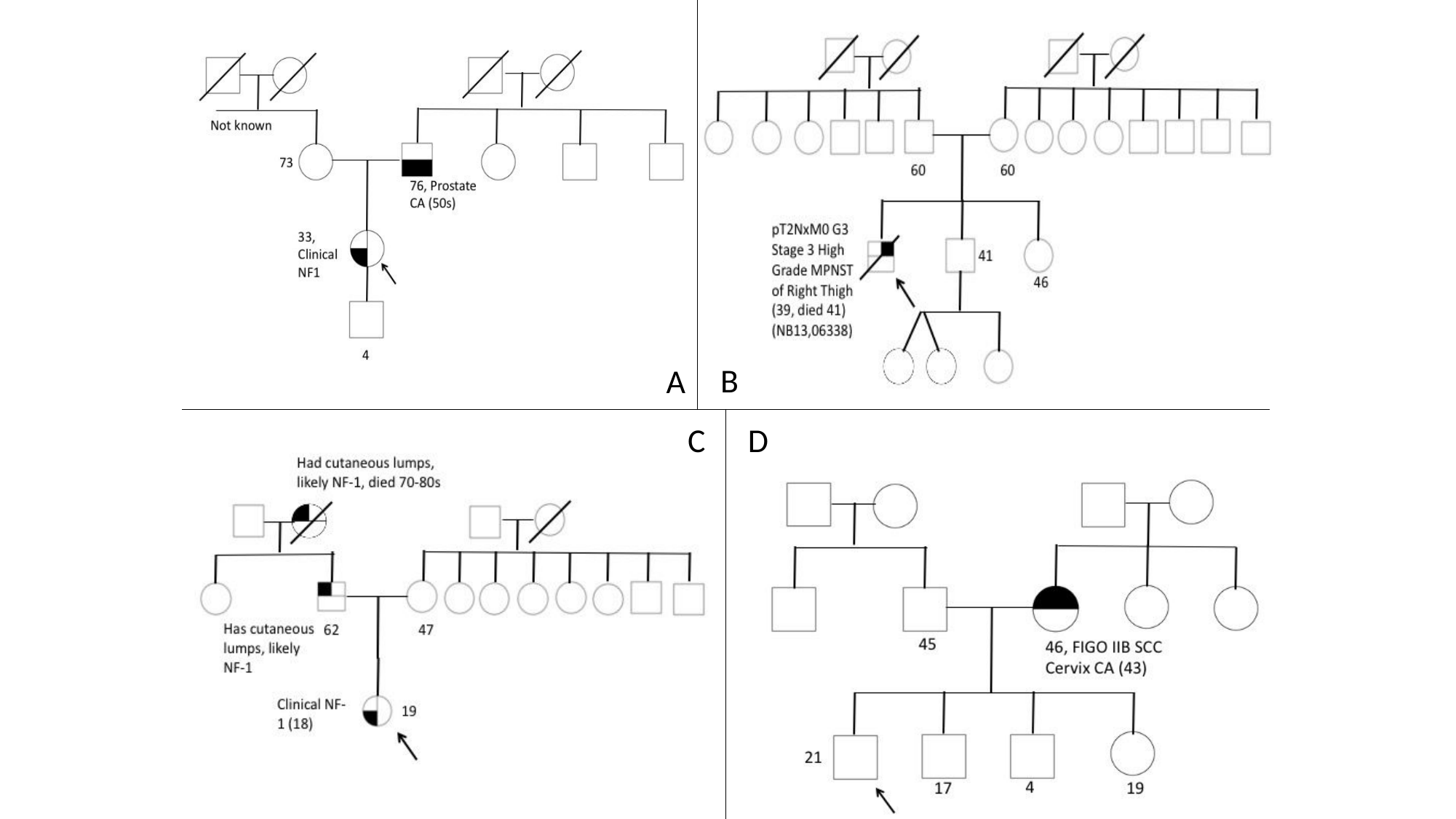

B
A
C
D

## Slide 4
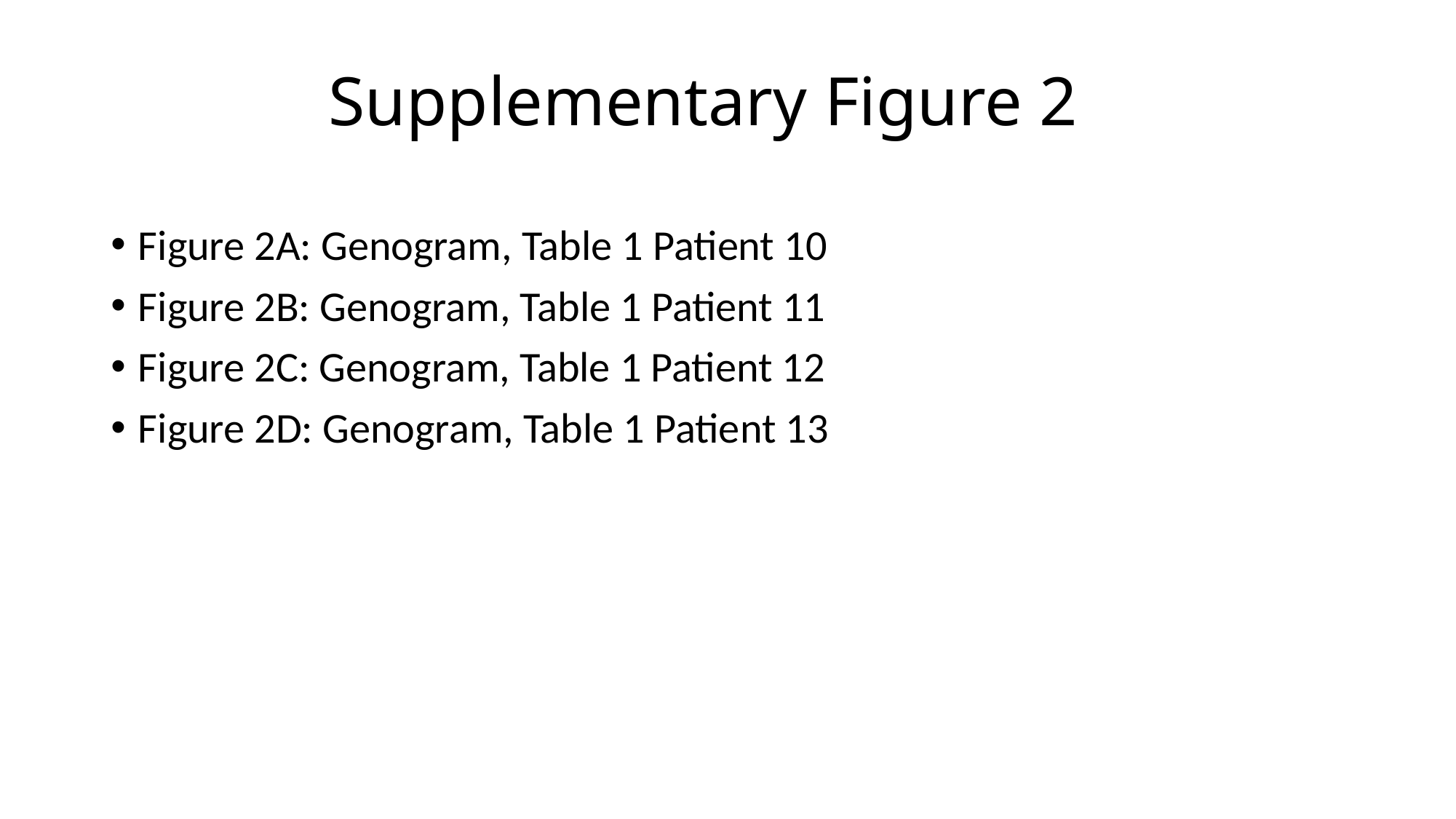

# Supplementary Figure 2
Figure 2A: Genogram, Table 1 Patient 10
Figure 2B: Genogram, Table 1 Patient 11
Figure 2C: Genogram, Table 1 Patient 12
Figure 2D: Genogram, Table 1 Patient 13
